# Alternative Antimicrobial Irrigation Strategies for the Treatment of Infections in Children: A Review of the Existing Literature

**DOI:** 10.3390/antibiotics12081271

**Published:** 2023-08-01

**Authors:** Costanza Di Chiara, Matteo Ponzoni, Pierre-Philippe Piché-Renaud, Daniele Mengato, Carlo Giaquinto, Shaun K. Morris, Daniele Donà

**Affiliations:** 1 Department for Women’s and Children’s Health, University of Padua, 35128 Padua, Italy; carlo.giaquinto@unipd.it (C.G.); daniele.dona@unipd.it (D.D.); 2 Penta—Child Health Research, 35127 Padua, Italy; 3 Division of Pediatric Infectious Diseases, The Hospital for Sick Children, Toronto, ON M5G 1X8, Canada; pp.piche-renaud@sickkids.ca (P.-P.P.-R.); shaun.morris@sickkids.ca (S.K.M.); 4 Child Health Evaluative Sciences, The Hospital for Sick Children, Toronto, ON M5G 1X8, Canada; 5 Congenital Cardiac Surgery Unit, Labatt Family Heart Centre, The Hospital for Sick Children, Toronto, ON M5G 1X8, Canada; matteo.ponzoni@sickkids.ca; 6 Hospital Pharmacy Department, University Hospital of Padua, Via Giustiniani, 2, 35128 Padua, Italy; daniele.mengato@aopd.veneto.it; 7 Department of Pediatrics, Faculty of Medicine, University of Toronto, Toronto, ON M5R 0A3, Canada; 8 Division of Clinical Public Health and Centre for Vaccine Preventable Diseases, Dalla Lana School of Public Health, Toronto, ON M5T 3M7, Canada

**Keywords:** antimicrobials irrigation, children, intra-cavity treatment, severe infections, review

## Abstract

As a synergistic treatment approach with systemic antimicrobial therapy or a systemic antibiotic-sparing strategy, the local administration of antimicrobial agents has been proposed as an alternative route for complicated infections. With the rationale of concentrating the active principle in the desired target site, avoiding potentially toxic systemic levels and bypassing anatomical and physiological barriers, local irrigation or infusion of antibiotics may effectively shorten the antimicrobial therapy course and reduce both infection-related and systemic therapy-related complications. Although evidence from the adult population supports its use in selected patients with an acceptable safety profile, data specifically focused on the pediatric population are limited. To provide a rapid and easily accessible tool for clinical practice, we synthesized the most relevant evidence on the use of local antimicrobial agents in common severe infections in children: meningitis, mediastinitis, pleural infections, recurrent urinary infections, and peritonitis. A literature search was performed using predefined combined keywords through an electronic research database (PubMed). Described molecules, dosages, routes, treated age groups, and related efficacy have been summarized for prompt application to clinical practice. It should, however, be noted that the evidence for the pediatric population remains limited, and the local administration of several molecules remains off-label. A careful multidisciplinary and patient-tailored evaluation, as well as a rational use of available guidelines, should always be the basis of clinical decision making in settings where local administration of antibiotics may be considered.

## 1. Introduction/State of the Art

Complicated infections, such as empyema, mediastinitis, peritonitis, and pyelonephritis, are uncommon but important causes of morbidity in children, often requiring long-term antimicrobial therapy courses and hospital stay [1]. Due to the low penetration of systemic antimicrobials in certain tissues or anatomical regions, increasingly caused by multi-drug resistant organisms (MRDOs), response to standard antimicrobial administration routes can be suboptimal or necessitate prolonged and multiple drug treatments, for certain infections [2]. Antimicrobial solution irrigation could thus represent an alternative or adjunctive strategy for the treatment of severe and complicated infections in select patients not responding to standard systemic therapy [3,4,5]. Local administration of antimicrobials may be an efficient strategy to optimize antibiotic use, since it allows for high antibiotic concentration directly at the site of infection. However, while antibiotic irrigations have been widely described for the prevention of surgical site infections both in children and adults, the safety and efficacy of antimicrobial intra-cavity infusion for the management of acute infections are poorly understood, and reports are mainly limited to adults.

In the present review, we synthesize the available evidence on the safety and effectiveness of antimicrobial irrigations for the treatment of certain complicated infections in pediatric populations. We conducted a targeted PubMed search, reviewing studies published until March 2023 that focused on the use of irrigation strategies for the management of pre-selected complicated infections in the pediatric population. Only articles written in English were considered. Additionally, we manually searched through the references. Our inclusion criteria encompassed case reports, case series, observational studies, clinical trials, and guidelines. Predefined search strings were used ((antimicrobials irrigations) OR (antimicrobials instillations) OR (antibiotics irrigations) OR (antifungal irrigations)) AND ((pediatric) OR (children) AND (meningitis and ventriculitis) OR (mediastinitis) OR (pleural infections) OR (peritonitis) OR (urinary tract infections) OR (empyema)).

**Figure 1:**
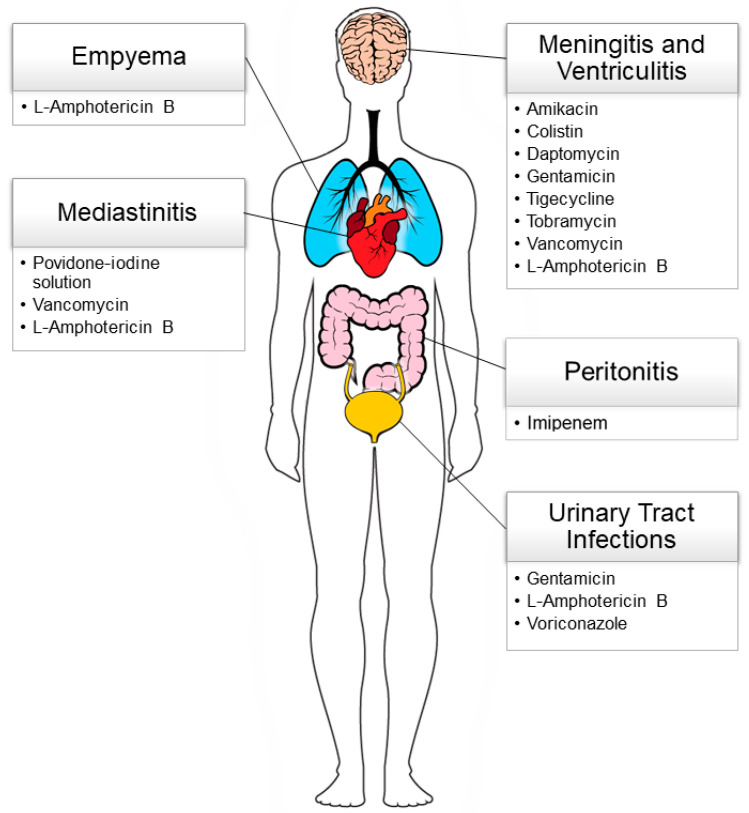
Schematic representation of the most common severe infections in the pediatric age where a local administration of antimicrobial agents has been described. The adopted active principles are reported.

To provide a valid and rapidly accessible tool to support clinical decision making, we summarized the antimicrobial formulations, doses, and timings for alternative local administration routes and their related efficacy and effectiveness that have been described in pediatric patients. Findings were organized in different paragraphs, according to the infections’ anatomical regions (Figure 1).

## 2. Results

### 2.1. Healthcare-Associated Meningitis and Ventriculitis

Healthcare-associated (HA) meningitis and ventriculitis are life-threatening infections occurring in approximately 3–15% of patients who undergo ventriculoperitoneal (VP) shunt placement [6,7]. Also named nosocomial central nervous system (CNS) infections, they can occur following severe craniocerebral trauma, neurosurgery, or intrathecal administration of cytotoxic drugs (i.e., intrathecal infusion pumps) and in patients with foreign body devices (i.e., internal and external cerebrospinal fluid shunts or drains). Moreover, prematurity, low birth weight (<2000 g), underlying diseases requiring invasive medical procedures (i.e., therapeutic lumbar puncture), and nosocomial bacteriemia are predisposing factors for the onset of HA ventriculitis and meningitis [8,9].

Methicillin-resistant coagulase-negative *Staphylococcus* spp., *Staphylococcus aureus*, including Methicillin-resistant *Staphylococcus aureus* (MRSA), vancomycin-resistant Enterococci, *Escherichia coli*, carbapenemase-producing Gram-negative bacteria (*Acinetobacter* spp., *Klebsiella* spp.), and *Pseudomonas aeruginosa* are the predominant organisms causing HA infections [8]. Although the majority of nosocomial CNS infections are bacterial, occasionally fungal VP shunt infection (e.g., *Candida* sp.) may occur [10].

Due to the high prevalence of MDROs, together with the presence of the blood–brain barrier that prevents an effective concentration of antibiotics in the cerebrospinal fluid (CSF) and their elevated incidence among children with underlying severe diseases (e.g., brain tumor, lymphoma, or lymphoblastic leukemia), HA meningitis is difficult to treat and can reoccur over time [11,12], leading to a high mortality rate [13].

Hence, the Infectious Diseases Society of America (IDSA) [14] recommended the intrathecal administration of antibacterial and antifungal agents “for patients with healthcare-associated ventriculitis and meningitis in which the infection responds poorly to systemic antimicrobial therapy alone”. This strategy should always be combined with appropriate systemic antibiotic therapy.

Several studies reported reassuring data on the intrathecal irrigation of antibiotics for the treatment of HA ventriculitis and meningitis. Recently, Nau et al. [15], reviewing the latest evidence on intrathecal antimicrobics infusion, described the efficacy and safety of this alternative drug administration route, mostly in adults. Aligned with IDSA recommendations, the authors highlighted that intrathecal antimicrobials irrigation should be considered in addition to systemic therapy, but should never be administered alone. Antimicrobials that are unable and/or too systemically toxic to reach a high concentration in the CSF after intravenous administration (such as vancomycin, teicoplanin, gentamicin, tobramycin, netilmicin, amikacin, streptomycin, colistin, polymyxin B, daptomycin, amphotericin B, and caspofungin, may be considered for intrathecal administration.

However, evidence on the use of intrathecal antimicrobials is mostly in adult populations. Data on the use of intrathecal antimicrobials infusion strategy in the pediatric population are limited to observational studies and case series.

Sahin et al. [16], among a cohort of 74 children (aged 16.7 ± 21.3 months) diagnosed with VP shunt infections, mainly caused by coagulase-negative *Staphylococcus* spp., carbapenem-resistant, and extended-spectrum-beta-lactamase (ESBL) positive Gram-negative bacteria, observed that children treated with both systemic and intrathecal antibiotics showed a faster clearance of CSF cultures (5.8 ± 2.6 vs. 11.5 ± 5.1 days, *p* < 0.05), and a shorter antibiotic course (19.2 ± 6.7 vs. and 26.6 ± 10.3 days, *p* < 0.05) and hospital stay (23.1 ± 6.4 vs. 36.3 ± 16.2 days, *p* < 0.05), compared to children receiving systemic therapy only. The most commonly used antibiotics for intrathecal irrigations were vancomycin (10 mg/day), amikacin (30 mg/day), and gentamicin (2 mg/day). In addition, colistin (1–4 mg/day), daptomycin (5 mg/day), and tigecycline (4 mg/day) were used in those patients infected by MDROs, according to the bacteria’s resistance profile. All patients underwent intra-ventricular drain removal and all antibiotic irrigations were performed via external ventricular tubes diluted in 5 mL of standard saline; then the tube was clamped for 1 h. No patients reported side effects related to the local administration of drugs, and all of them recovered from VP shunt infection.

Similarly, Aarnivala et al. [17] documented the use of intrathecal tobramycin at 5 mg/day in a 2-year-old immunocompromised boy affected by disseminated *P. aeruginosa* MDR infection during chemotherapy. Due to the failure of intravenous antibiotic therapy with meropenem and tobramycin, the child was treated with intrathecal antibiotic irrigations, with his clinical condition improving after three days of treatment and without any adverse effects.

Conversely, Xiao et al. [18], in nine highly compromised children diagnosed with *A. baumannii* MDR (susceptible to amikacin), extensively drug-resistant (susceptible to polymyxin and tigecyclin), and pan-drug-resistant (tigecycline intermediate) meningitis, observed poor outcomes in those patients who were treated with intrathecal amikacin (10–15 mg/day). Two out of three children who underwent intrathecal irrigation died, and there were no significant differences in the time to clearance of CSF cultures. However, these findings should be interpreted in light of the difficulty of eradicating *A. baumannii* MDR per se, which is known to affect patients with multiple comorbidities, independently from the route of administration of antibiotics.

Nakwan et al. [19] reviewed 17 studies investigating the use of colistin in a total of 312 neonates and infants affected by MDRO severe infections (mainly caused by *A. baumannii*, *P. aeruginosa*, *K. pneumonia*, and *E. coli*). Colistin was administered intrathecally in seven patients, who were treated with a dose between 20,000 IU/kg/day (=1.6 mg of colistimethate/kg/day) and 125,000 IU/day (=10 mg of colistimethate/day). All patients survived, and no adverse events were described in patients who received intrathecal colistin.

Al Yazidi et al. [20] also presented a case of *E. cloacae* meningitis in a preterm male neurosurgical patient who was successfully treated with intrathecal colistin in a dose of 5 mg/day, diluted in 2 mL of saline, combined with systemic antibiotic treatment. The ventricular tube was clamped for one hour and then released. CSF cultures cleared only 24 h after the beginning of intrathecal infusions, and the infant received a total of nine infusions without any adverse events.

Although fungi cause meningitis less commonly than bacteria, fungal meningitis may occur in fragile newborns, owing to prematurity, low birth weight, or prolonged parenteral nutrition.

Yuan et al. [21] described a case of relapsing *Candida tropicalis* meningitis in a 6-month-old girl that was treated with first systemic fluconazole for one month and which was shifted to systemic voriconazole without achieving the plasma and CSF target therapeutic concentrations. Due to systemic therapy failure, voriconazole was shifted to systemic liposomal amphotericin B (L-amphotericin B). However, due to the poor ability of L-amphotericin B to cross the blood–brain barrier, intrathecal L-amphotericin B therapy was introduced as a rescue strategy at a starting dose of 0.025 mg, which was increased by 0.025 mg every 2 days. After 7 days of treatment, the infant improved clinically. Due to renal failure 25 days later, the systemic L-amphotericin B was discontinued, and the intrathecal irrigations were maintained for a total of 2 months. After the 1-year follow-up, the infant had fully recovered, without sequelae. This case highlights another potential application of the local antimicrobial treatment as an option to reduce the side effects of systemic administration while maintaining a highly effective concentration of the active agent at the desired site.

Similarly, Bhatti et al. [22] reported on the cases of a 4-month-old female infant and a 5-month-old male infant with VP shunt-related meningitis due to azole-sensitive *C. albicans*. The two infants were treated with intrathecal L-amphotericin B irrigations combined with systemic therapy, without adverse events. Moreover, Murphy et al. [23] reported two cases of *C. albicans* VP shunt-related meningitis in 2-year-old children, who were successfully treated with 1 mg/day of intraventricular L-amphotericin B for 11 and 7 days, respectively, combined with systemic treatment. Finally, a 3-year-old immunocompromised girl affected by right rhino-orbital-cerebral mucormycosis caused by *Lichtheimia corymbifera* [24] was extensively treated for a total of six weeks with systemic L-amphotericin-B, posaconazole, and terbinafine as, well as with profound intra- and extra-cranial surgical debridement and hyperbaric oxygen therapy. Due to the presence of a severe and invasive infection in a high-risk patient, she underwent 114 intrathecal irrigations of L-amphotericin-B (0.5 mg/day, 5 mg/mL sterile water solution) via the Ommaya reservoir, with no notable neurotoxicity. The patient’s condition improved, and she underwent reconstructive surgery and was discharged after ten months of hospitalization.

In conclusion, although intrathecal irrigation of antimicrobials is not routinely recommended for HA-meningitis and ventriculitis in the pediatric population, intrathecal administration of antibiotics and antifungals may be considered as a rescue treatment in selected cases when systemic treatment options fail or lead to important side effects. Finally, as mentioned above, the intrathecal antimicrobial administration seems effective in reducing the total duration of antibiotic therapy, representing a potential strategy to decrease antimicrobial resistance and hospitalization costs. Table 1 summarizes antimicrobial formulations and doses for intrathecal irrigations in children.

### 2.2. Mediastinitis

Infective mediastinitis (IM) is an extremely rare condition in the pediatric population, but some studies have reported that it could occur as a complication of cardiac surgery in approximately 1–3% of procedures [25,26,27]. Delayed sternal closure, a common practice in neonatal or complex surgeries, seems to increase the incidence of mediastinitis in up to 4–5% of operations, but the evidence is derived from small case series, and a proven increased risk is still controversial [25]. In this setting, IM could complicate the postoperative course dramatically, leading to the patient’s death in 10–20% of cases [25,26,27,28].

There are still no globally accepted standardized management protocols for IM [21]; however, there are some retrospective series describing successful management, where post-sternotomy IM is treated with a combined surgical and medical approach. In particular, surgical debridement of necrotic or infected soft and bone tissues is a cornerstone of treatment, followed by intra-mediastinal irrigation with povidone-iodine solution [27] or antibiotics through chest tubes [26], which are left in place for a mean of 8–15 days until culture clearance, depending on the series [26,27].

The most commonly used drug for local administration in IM is vancomycin, although some authors describe intra-mediastinal irrigation with amphotericin B in anecdotal cases of fungal mediastinitis [26]. Reported dosages are summarized in Table 2.

There have been some concerns around the systemic absorption of intra-mediastinal iodine, which has been observed in an experimental study [29], although it has not led to a significant alteration in thyroid function in a report of 18 children by Kovacikova et al. [30]. The short follow-up (2 weeks after discontinuation of mediastinal irrigation), and thus this study, cannot make inferences about long-term sequelae. Furthermore, the povidone-iodine solution usually needs high-volume irrigations, which could affect the hemodynamics of smaller patients. On the other hand, the systemic absorption of gentamicin has been demonstrated after local irrigation in post-sternotomy IM in adults [31], especially in patients with smaller body surface area. Thus, monitoring serum antibiotic concentrations, as well as signs of renal or liver toxicity, is mandatory.

**Table 2:** Reported dosing and intra-mediastinal irrigation solutions for the treatment of mediastinitis in the pediatric population.

Active Principle	Dosage and Solution	Treated Age Class	Tips for Infusion	Reference
Povidone-iodine solution	5–10 mL of 10% solution/1000 mL saline	Infants and children < 5 years	100–200 mL/h	Ugaki et al. [27]
	0.05–0.005% solution	Infants and children < 5 years	20 mL/h	Kovacikova et al. [30]
Vancomycin	500 mg/1000 mL 0.9% saline	Children > 2 months (2 months–10 years)	20 mL/h	Vida et al. [26]
L-Amphotericin B	25 mg/100 mL 0.9% saline	Children > 2 months (2 months–10 years)	20 mL/h	Vida et al. [26]

Finally, the local application of antimicrobial agents in the surgical field can be considered in cases of active endocarditis or cardiac abscess [32]. Although the administration of minocycline powder on infected intracardiac structures and on a newly implanted organ has been described in adults with good efficacy and safety profiles [33], this technique has never been used in the pediatric population.

Although our review has not identified any randomized controlled trials on the safety and efficacy of intra-mediastinal irrigation with antibiotics, based on available evidence, this technique may be considered for supporting surgical debridement and targeted systemic antimicrobial therapy in children with IM following cardiac surgery, on a case-by-case basis. To minimize systemic toxicity in the absence of sufficient data about their long-term safety, povidone-iodine irrigations should be used carefully in the pediatric population, and systemic levels of potential absorbable antibiotics should be frequently monitored.

### 2.3. Pleural Infections

Pleural infections (PIs), including complicated parapneumonic pleural effusions and pleural parapneumonic empyema, can develop in 2–12% of pneumonia in children, requiring hospitalization in up to 28% of cases [34]. The parapneumonic effusion consists of “pleural fluid collection in association with underlying pneumonia”, while empyema is defined as “the presence of pus in the pleural space” [35]. Their formation is a progressive process distinguished by three stages: parapneumonic effusion (Stage 1), an uncomplicated free-flowing parapneumonic effusion not containing bacterial organisms; the fibrinopurulent stage (Stage 2), with a bacterial invasion across the damaged lung epithelium that, stimulating the immune response, supports fibrin deposition (loculated effusion) and pus formation (empyema stage); and the chronic organizing stage (Stage 3), when a solid fibrous pleural cortex is present [36].

The most common bacterial pathogens causing PI are *Streptococcus pneumonia* and *Staphylococcus aureus*, including MRSA, followed by *group A Streptococci* (e.g., *Streptococcus pyogenes*) [34]. Parapneumonic effusions may also be caused by *Mycobacterium tuberculosis* and fungi (e.g., *Aspergillus* spp.) [34].

As reported by the British Thoracic Society [35], the cornerstones of the treatment for PIs are prompt initiation of an appropriate empirical antimicrobial therapy covering the most frequent organisms involved in community-acquired or nosocomial pneumonia if no organisms are identified, combined with the surgical evacuation of the infected collection from the pleural space. However, several factors may reduce antibiotic efficacy in the pleural cavity, and thus fibrinolytic therapy or surgical debridement of the pleural space could be required in certain complicated cases.

While the penetration of antibiotics into the pleural space has been demonstrated to be satisfactory in animal models [37], human studies on the intra-pleural concentration of systemic antibiotics are still lacking. In addition, loculated empyema can impede the sterilization of the entire pleural cavity by systemic antimicrobial therapy.

Intrapleural instillation of antibiotics may be an attractive alternative to overcome lower penetration of antimicrobial agents in the pleural space when septate or loculated effusions are present, aiming at achieving a more homogeneous distribution of the drugs into the pleural cavity. However, the evidence of efficacy, safety, and tolerability of this procedure is scarce, limited to adults or animal models, and relies mostly on old retrospective case series [38,39]. To date, no data are available for the pediatric population.

Recently, Torbic et al. [40], conducted a study on different management strategies, including intrapleural antibiotic irrigation for post-pneumonectomy empyema, in 18 adults (median age of 68 years old (interquartile range (IQR): 56–78)) affected by lung cancer. All patients received intrapleural antibiotic irrigations after six days of broad-spectrum systemic antimicrobial therapy. The most common intrapleural antibiotic prescribed was vancomycin (94% of patients). Other intrapleural antimicrobials that were employed included ceftazidime, meropenem, voriconazole, and metronidazole. Antibiotic dilutions are listed in Table 3. The median number of intrapleural antimicrobial irrigations received per patient was 5.5 (IQR: 1–9). Intrapleural antibiotics were administered at 42 mL/h via a chest tube, or as a bolus, once per day. The antibiotic was left in the pleural cavity for a median of 6.3 h [IQR: 4–10]) and then drained. No adverse events were reported, suggesting that the intrapleural antibiotic instillation was well tolerated in this study.

There is limited evidence on the use of intrapleural antifungals in children, with only a few case reports published on the treatment of invasive pulmonary aspergillosis. Baquero-Artigao et al. [41] and Almuhareb et al. [42] treated with combined systemic and intrapleural antifungals an empyema caused by *Aspergillus fumigatus* in two 12-year-old and 8-year-old immunocompromised boys. In particular, the first patient [41], after debridement surgery and systemic administration of L-amphotericin B, developed pulmonary empyema and a bronchopleural-cutaneous fistula. He was treated with 5 mg of L-amphotericin B diluted in 10 mL of dextrose 5%, infused via a chest tube for 5 min as an initial dose, which was gradually increased to 50 mg/day. After the infusion, the catheter was clamped and the solution remained in the pleural cavity for 30 min. The child’s condition improved, and cultures became negative within 2 weeks of local treatment. Pleural infusions were continued for a total of 45 days, and then the systemic therapy was maintained alone for a total of 6 months of treatment. No adverse drug reactions were reported during antifungal intra-cavity irrigations.

Almuhareb et al. [42] adopted combined systemic voriconazole and caspofungin and L-amphotericin B intrapleural irrigations, in addition to surgical intervention. In this case, the authors administered 5 mg/kg of L-amphotericin B diluted in 50 mL of dextrose 5% into a pleural space via a chest tube for 30 min, once daily; the solution was left in the cavity for 2 h and then the tube was declamped. The intrapleural therapy was continued for 6 weeks, combined with systemic antifungals; the child improved after 10 days, without complications.

Although intrapleural irrigation has the potential for bypassing the poor pleural penetrance of certain antimicrobial agents and supporting the systemic therapy of complicated parapneumonic pleural effusions, data related to this technique are limited to case reports of children with a rare fungal infection. To our knowledge, no pediatric cases have been documented of the successful use of this technique to treat bacterial infections. Therefore, the routine use of intrapleural irrigations has not been included in any pediatric guidelines. Before being considered as an additional line of treatment, further studies are needed to assess the efficacy of this practice in a larger subset of patients, including children and adolescents.

### 2.4. Recurrent Urinary Tract Infections

Recurrent urinary tract infections (UTIs) are relatively common in children, with significant impacts on their quality of life, sometimes resulting in more serious morbidities (e.g., pyelonephritis or urosepsis). Children with predisposing anatomical and/or functional conditions, such as genitourinary malformations, vesicoureteral reflux, and dysfunction [43] are the most frequently affected population. In addition, recurrent UTIs often require repeated courses of oral or intravenous antibiotics, contributing to the emergence of MDROs, including beta-lactamase (ESBLs and carbapenemases) producers [44]. Furthermore, multiple antibiotic courses may turn into an additional risk factor for fungal infections and local and/or systemic complications when an underlying genitourinary organic disease is present.

To address the challenging clinical management of such patients, since the 1960s, several authors have proposed intravesical antimicrobial instillation as a valid and effective strategy for treating recurrent UTIs and renal fungal infections in the pediatric population [45,46].

More recently, Defoor et al. [47] retrospectively described 80 children (median age 10 years) with neuropathic bladder, and they underwent gentamicin intravesical irrigations for symptomatic recurrent UTIs or prophylaxis during invasive procedures, reporting encouraging results. No serum gentamicin levels greater than 0.4 g/mL (therapeutic trough level 0.2 g/mL to 2 g/mL) were detected, nor adverse events, and neither additional discomfort nor secondary fungal infections were documented during the treatment.

Similar results have been recently obtained by Marei et al. [48]. In a cohort of 24 children (median age 3.8 years) with urologic comorbidities, gentamicin intravesical installations (see Table 4 for dosage) were well tolerated and safe for treating (14/24) or preventing (10/24) urinary tract infections. Among 14 children with UTIs, 12/14 recovered after a 7-day treatment regimen. In addition, 58% of patients on a prophylactic regimen were free from breakthrough UTIs for 252 days (median: 256 days, IQR: 85–352). Intravesical gentamicin was administered at a dose of 8 mg in 20 mL or 20 mg in 50 mL of standard saline once or twice per day, depending on bladder capacity and the therapeutic or prophylactic regimen, respectively. Neither the detection of serum gentamicin nor adverse events secondary to the intravesical instillation were reported. Finally, gentamicin resistance emerged in only one case (4.2%), an ESBL/AmpC-producing *Escherichia coli*.

Two additional studies enrolling pediatric patients documented satisfactory results of the prophylactic intravesical irrigation of antibiotics to reduce the incidence of recurrent UTIs with MDROs. Huen et al. [49], with a cohort of 52 children (median age of 14.5 years) affected by neurogenic bladder necessitating clean intermittent catheterization, administered prophylactic neomycin-polymyxin or gentamicin intravesical installations. The authors observed a decrease in symptomatic recurrent UTIs (incidence rate ratio (IRR): 0.42, 95% confidence interval (CI): 0.31–0.56; *p* < 0.001) and patients’ hospitalization (IRR: 0.61, 95% CI: 0.37–0.98; *p* = 0.043). Moreover, they also observed a reduction in MDRO isolations from urine cultures. Comparable results were reported by Cox et al. [50].

Although the level of evidence regarding the use of antimicrobial intravesical instillation for the treatment of UTIs in pediatric patients is supported by observational studies with small sample sizes, reassuring data from larger studies and systematic reviews can be derived from the adult population. Pietropaolo et al. [51], reviewing 11 studies including a total of 285 adult patients treated with antibiotic intravesical irrigations (117 for UTI treatment and 168 for prophylaxis), proved a significant reduction in symptomatic UTIs in 78% of cases, a low rate of minor complications, and a 23–30% reduction in antibiotic resistance of microorganisms, allowing the eradication of resistant germs or an earlier shift to oral antibiotics.

Similarly, data on intravesical antifungal irrigations is mainly confined to adults. Local administration of L-amphotericin B (50–100 mg/L of sterile water), combined with systemic and surgical treatments, is recommended for the management of renal and/or ureteral fungal balls by the Infectious Diseases Society of America [52]. Alternative antifungal agents described for nephrostomy tube irrigations in adults include fluconazole, anidulafungin, and caspofungin [53,54]. Moreover, deoxycholate amphotericin B at 50 mg/L of sterile water has also been considered in the treatment of lower UTIs, due to fluconazole-resistant *Candida* spp. [52].

**Table 4:** Dosing of the most commonly used intravesical antimicrobial agents in the treatment of complicated urinary tract infections in the pediatric population.

Active Principle	Dosage and Solution	Treated Age Class	Tips for Infusion	Reference
Antibiotics				
Gentamicin	8 mg in 20 mL or 20 mg in 50 mL of 0.9% saline once (if prophylactic regimen) or twice (if therapeutic regimen) per day	Children >1 year and adults	intra-vesical installations	Marei et al. [48]
Antifungals				
L-amphotericin B	5 mL of the 0.05 mg/mL solution every 6 h	Low-birth-weight preterms	intra-nephrostomy infusions	Chen et al. [55]
Voriconazole	200 mg diluted in 1 L of 0.9% saline			González-Vicent et al. [56]

Few case reports or short series describe the use of antifungal bladder or nephrostomy irrigations in children. Chen et al. [55] reviewed the course of seven very-low-birth-weight preterm infants who were treated with L-amphotericin B intra-nephrostomy infusions for obstructive renal candidiasis in addition to surgical intervention and systemic therapy, suggesting that antifungal irrigation may be an important adjunctive therapy to fragment fungal balls, facilitate the elimination of debris, and prevent fibrin occlusion of the nephrostomy also in neonates. Due to the absence of specific guidelines, the authors adopted a regimen of 5 mL L-amphotericin B (0.05 mg/mL) infused every 6 h for 30 days (Table 4), until the normalization of urinalysis, the achievement of negative urine cultures, and the radiologic resolution of fungal balls.

A single case report describes intravesical antifungal irrigation in an immunocompromised child who developed disseminated aspergillosis with an intravesical fungus ball [56]. The child was treated with systemic antifungals in combination with the intravesical instillation of voriconazole for 6 days (200 mg diluted in 1 L of saline once daily), with a good response to the bladder lesion. However, the child also necessitated surgical treatment, due to the large dimensions of the fungal ball.

In conclusion, intravesical irrigations of antimicrobials (especially antibiotics) seem to be effective and safe in treating and preventing infections in children with genito-urinary tract comorbidities. In the absence of systemic signs of infection, intravesical irrigation alone could represent an antibiotic-sparing strategy to treat recurrent UTIs. Moreover, by reducing the hospitalization rate and making the self-administration of the drug at home easier, antibiotic bladder irrigations could improve the quality of life of children with renal and urinary malformations and/or dysfunction. Despite this, the data remain confined to small observational studies, with a low level of evidence to inform recommendations in children.

### 2.5. Peritonitis and Intra-Abdominal Abscesses

Intra-abdominal infections (IAIs) are one of the most common postoperative complications in children treated for perforated appendicitis [57,58,59,60,61]. The cornerstone of IAI treatment is adequate source control, as well as antibiotic therapy, which is essential to prevent and minimize further complications, and improve patients’ outcomes. In addition, source control can effectively shorten the course of antibiotic therapy, which could in turn mitigate the development of antimicrobial resistance.

The goal of the source control strategy is the total elimination of any infective focus, such as draining pus from abscesses and washing out necrotic tissues and cell debrides. Therefore, an extensive lavage of the abdominal cavity has been considered crucial to optimize the outcomes of source control procedures in generalized peritonitis. Irrigation with saline solution and aspiration are both described as effective in removing cell debris and infective material contained in the abdominal cavity, both in children and adults [62,63,64]. In addition to the standard washout procedure with saline solution, peritoneal irrigations with antibiotic agents have been proposed as an adjunctive strategy to achieve a more extensive lavage of the abdomen, contributing to infection source control. In the past decades, different antibiotics and antiseptic agents have been proposed for abdomen irrigations, but the evidence of their efficacy compared to standard instillation with saline solution is limited and remains controversial [65].

Recently, Raeiszadeh et al. [66], performed a randomized clinical trial evaluating the efficacy of abdominal lavage with standard saline and gentamicin versus saline only, in a cohort of 80 adults who underwent an urgent laparotomy for acute peritonitis. Noticeably, the addition of gentamicin almost halved the rate of surgical interventions needed, compared to the control group (17.5% vs. 35%, *p* = 0.039).

Similarly, Hesami et al. [67] conducted a randomized single-blinded clinical trial on 90 subjects (aged 12–50 years), who underwent emergent surgery due to acute abdomen. Patients were randomly allocated into two treatment groups: abdominal irrigation with normal saline plus imipenem (1 mg/mL) vs. normal saline only (control group). All patients received intravenous ampicillin-sulbactam (3 g, 4 times a day), starting 30 min before the surgery. The authors observed a significantly higher rate of postoperative infective complications in the control group, compared to the experimental one (22% vs. 4%, respectively, *p* = 0.013). Specifically, patients who received standard irrigations reported a higher incidence of surgical wound infection (11% vs. 4%, *p* = 0.23) and abdominal abscess (13% vs. 2%, *p* = 0.049). Moreover, the total hospital stay was significantly shorter in the treatment group (4.9 vs. 5.8 days, *p* = 0.034).

A similar prospective study performed by Santhosh C. S. et al. [68] 4 years later aligns with previous findings. In a cohort of 90 subjects (aged 12–60 years) admitted to a tertiary-care teaching hospital in Bangalore for complicated peritonitis, the authors evaluated the efficacy of intra-peritoneal irrigations with imipenem in reducing postoperative morbidities. Patients were randomly and equally assigned to three different treatment groups: abdominal irrigation with saline solution, followed by fluid draining (group 1), normal saline plus imipenem (1 mg/mL) irrigation, followed by liquid evacuation after 5 min (group 2), and normal saline plus imipenem (1 mg/mL) irrigation, followed by liquid evacuation after one hour (group 3). A significant reduction in the incidence of surgical wound infections (50% vs. 33% vs. 17% in group 1, 2, and 3, respectively, *p* = 0.023), intra-abdominal abscess (30% vs. 10% vs. 7% in group 1, 2, and 3, respectively, *p* = 0.026), sepsis (30% vs. 10% vs. 7% in group 1, 2, and 3, respectively, *p* = 0.026), fecal fistula (17% vs. 10% vs. 0, in group 1, 2, and 3, respectively, *p* = 0.07), re-laparotomy (20% vs. 0 vs. 0, in group 1, 2, and 3, respectively, *p* = 0.001), and death (17% vs. 7% vs. 3%, in group 1, 2, and 3, respectively, *p* = 0.09) was observed with the imipenem irrigations.

Table 5 summarizes antimicrobial formulations and doses for intra-abdominal irrigations in children.

Although these findings support the fact that peritoneal irrigations with antibiotic solutions may serve as a safe tool to reduce post-operative morbidity and shorten hospitalization times and the duration of systemic antibiotic treatment in adult patients with peritonitis, their safety and efficacy profiles have not been specifically addressed in the pediatric population. While previous studies observed comparable outcomes for either peritoneal lavage with saline or suction, in the setting of complicated appendicitis in children [61,63], reports investigating the use of intra-abdominal irrigations with antibiotics in the pediatric age group are still lacking. Moreover, despite the availability of randomized controlled trials enrolling mixed pediatric–adult cohorts (see above), their sample size is too small to allow for the safe generalization of findings, especially in younger patients. Novel insights into the efficacy of intra-abdominal administration of antibiotics are expected from an ongoing clinical trial, specifically designed to analyze the clinical impact of a high antibiotic concentration provided through intra-abdominal irrigations (gentamicin and/or clindamycin) compared to normal saline, in patients with abdominal abscess (available at: https://ichgcp.net/clinical-trials-registry/NCT03476941; accessed on 1 March 2023).

## 3. Limitations

Although the scientific literature was extensively reviewed, the present study is not a systematic review, and thus other reviews might become available to systematically describe the local administration of antibiotics in the pediatric age group. As stated in the introduction, we focused on the most significant clinical scenarios where a topical use of antimicrobial agents has been described. For more uncommon conditions, we suggest a careful and targeted investigation of the scientific literature. With the present work, we aimed to summarize and collate the most relevant evidence on alternative ways of administration of antibiotics in challenging clinical settings, to provide a rapid and easily accessible tool for physicians. For this purpose, antimicrobial agents, dosages, and treated populations have been listed in dedicated tables, which should be used to ease the literature review and do not represent a clinical indication or a recommendation. Finally, we want to underline the fact that several of the cited studies entail an off-label use of antimicrobial agents and refer to studies of low-grade quality (Table 6), whose adoption should be considered after multidisciplinary evaluation and dedicated ethics board review and approval, when deemed necessary.

## 4. Conclusions

The local administration of antimicrobial agents in complicated infections has several advantages. This strategy conveys the active molecules to the target site, achieving high concentrations without the need for potentially toxic systemic levels, and it can bypass anatomical and physiological barriers to an efficient distribution of antimicrobials. Its clinical translation, although mainly supported by small retrospective studies or case series in the pediatric population, is a systemic antibiotic-sparing approach that may shorten the antimicrobial therapy course and hospitalization times, containing the local complications and systemic side-effects of the therapy itself. In the present work, we synthesized the most relevant evidence on the use of local antimicrobial agents in common clinical scenarios that physicians may encounter when treating severe infections in children. Aiming at providing a ready-to-use tool for clinical practice, we summarized the reported molecules, dosages, and treated cohorts, together with their efficacy, and described safety concerns.

## Figures and Tables

**Table 1:** Reported antimicrobial agents for the local treatment of central nervous system infections in the pediatric population. The dose of intraventricular antibiotics for children can be also calculated by reducing the amount used for adult patients by 60%, in line with IDSA recommendations [14].

Active Principle	Dosage and Solution	Treated Age Class	Tips for Infusion	Reference
Antibiotics				
Amikacin	30 mg/day in 5 mL of 0.9% saline	Children > 1 year	The external ventricular tube was clamped for 1 h and then released.	Sahin et al. [16]
Colistin	1–4 mg/day in 5 mL of 0.9% saline	Children > 1 year	The external ventricular tube was clamped for 1 h and then released.	Sahin et al. [16]
	20,000 IU/kg/day to a maximum of 125,000 IU/day	Neonates and infants < 1 year		Nakwan et al. [19]
	5 mg/day diluted in 2 mL of 0.9% saline	Preterm	The ventricular tube was clamped for 1 h and then released.	Al Yazidi et al. [20]
Daptomycin	5 mg/day in 5 mL of 0.9% saline	Children > 1 year	The external ventricular tube was clamped for 1 h and then released.	Sahin et al. [16]
Gentamicin	2 mg/day in 5 mL of 0.9% saline	Children > 1 year	The external ventricular tube was clamped for 1 h and then released.	Sahin et al. [16]
Tigecycline	4 mg/day in 5 mL of 0.9% saline	Children > 1 year	The external ventricular tube was clamped for 1 h and then released.	Sahin et al. [16]
Tobramycin	5 mg/day	Children > 1 year		Aarnivala et al. [17]
Vancomycin	10 mg/day diluted in 5 mL of 0.9% saline	Children > 1 year	The external ventricular tube was clamped for 1 h and then released.	Sahin et al. [16]
Antifungals				
L-Amphotericin B	starting dose of 0.025 mg/day which was increased by 0.025 mg/day for a total of 7 days,	Neonate		Yuan et al. [21]
	0.5 mg/day, 5 mg/mL of sterile water solution	Children > 1 year	Ommaya reservoir was used for the infusion.	Jensen et al. [24]

**Table 3:** Reported dosing for intrapleural irrigation solutions for the treatment of empyema in the pediatric population. Data on intrapleural antimicrobial irrigation in children are limited to case reports focusing only on aspergillosis.

Active Principle	Dosage and Solution	Treated Age Class	Tips for Infusion	Reference
L-Amphotericin B	starting dose 5 mg/day then gradually increased to 50 mg/day, diluted in 10 mL of dextrose 5%	Children > 1 year	the catheter was clamped and the solution remained in the pleural cavity for 30 min	Baquero-Artigao et al. [41]
	5 mg/kg/day diluted in 50 mL of dextrose 5%	Children > 1 year	the catheter was clamped and the solution remained in the pleural cavity for 2 h	Almuhareb et al. [42]

**Table 5:** Dosing of the most commonly used intra-abdominal antimicrobial agents in the treatment of complicated peritonitis in the pediatric population.

Active Principle	Dosage and Solution	Treated Age Class	Tips for Infusion	Reference
Imipenem	1 mg/mL of 0.9% saline	Children > 1 year and adults		Hesami et al. [67]
	1 mg/mL of 0.9% saline	Adolescents > 12 years and adults	the catheter was clamped and the solution remained in the pleural cavity for 1 h	Santhosh C. S. et al. [68]

**Table 6:** Summary of level of evidence for antimicrobial irrigations for infections occurring in different anatomical regions.

Type of Infection/Anatomical Region	Level of Evidence in Adults	Level of Evidence in Children
Meningitis and ventriculitis	Review articles and observational studies	Observational studies, case series, and case reports
Mediastinitis	Cohort studies and case series	Case series and case reports
Pleural infections	Case series	Case reports
Urinary tract infections	Cohort studies, case series, and case reports	Cohort studies, case series, and case reports
Peritonitis and intra-abdominal abscesses	Single-blinded clinical trial, cohort study	Cohort study (clinical trials are ongoing)

## Data Availability

Articles included in this review are available through Pubmed database using the following predefined search strings: ((antimicrobials irrigations) OR (antimicrobials instillations) OR (antibiotics irrigations) OR (antifungal irrigations)) AND ((pediatric) OR (children) AND (meningitis and ventriculitis) OR (mediastinitis) OR (pleural infections) OR (peritonitis) OR (urinary tract infections) OR (empyema)).
